# Guest and solvent modulated photo-driven charge separation and triplet generation in a perylene bisimide cyclophane†
†Electronic supplementary information (ESI) available: Experimental details including steady-state and transient absorption spectera as described in the text. See DOI: 10.1039/c6sc01574c


**DOI:** 10.1039/c6sc01574c

**Published:** 2016-05-18

**Authors:** Peter Spenst, Ryan M. Young, Michael R. Wasielewski, Frank Würthner

**Affiliations:** a Institut für Organische Chemie and Center for Nanosystems Chemistry , Universität Würzburg , Am Hubland , 97074 Würzburg , Germany . Email: wuerthner@chemie.uni-wuerzburg.de; b Department of Chemistry and Argonne-Northwestern Solar Energy Research (ANSER) Center , Northwestern University , 2145 Sheridan Road , Evanston , IL 60208-3113 , USA . Email: m-wasielewski@northwestern.edu

## Abstract

Cofacial positioning of two perylene bisimide (PBI) chromophores at a distance of 6.5 Å in a cyclophane structure prohibits the otherwise common excimer formation and directs photoexcited singlet state relaxation towards intramolecular symmetry-breaking charge separation (*τ*_CS_ = 161 ± 4 ps) in polar CH_2_Cl_2_, which is thermodynamically favored with a Gibbs free energy of Δ*G*_CS_ = –0.32 eV. The charges then recombine slowly in *τ*_CR_ = 8.90 ± 0.06 ns to form the PBI triplet excited state, which can be used subsequently to generate singlet oxygen in 27% quantum yield. This sequence of events is eliminated by dissolving the PBI cyclophane in non-polar toluene, where only excited singlet state decay occurs. In contrast, complexation of electron-rich aromatic hydrocarbons by the host PBI cyclophane followed by photoexcitation of PBI results in ultrafast electron transfer (<10 ps) from the guest to the PBI in CH_2_Cl_2_. The rate constants for charge separation and recombination increase as the guest molecules become easier to oxidize, demonstrating that charge separation occurs close to the peak of the Marcus curve and the recombination lies far into the Marcus inverted region.

## Abstract 2

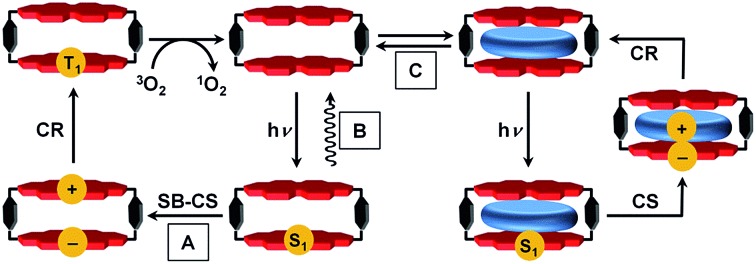
The modulation of the excited state dynamics in a perylene bisimide cyclophane was achieved by solvent and guest variation.

## Introduction

The precise positioning of the light absorbing chlorophylls and their associated redox cofactors in photosynthetic reaction center proteins using weak metal–ligand and hydrogen bonds results in optimized electronic interactions between them that result in efficient charge separation to form radical ion pairs (RPs).^1^–^4^ For example, photoexcitation of the chlorophyll special pair dimer in some reaction center proteins leads to symmetry-breaking charge separation.^5^ There is a long and rich history involving the design, synthesis, and characterization of covalent electron donor–acceptor systems that model the electron transfer (ET) processes within reaction center proteins.^6^–^12^ In general, however, efforts to employ non-covalent molecular interactions and self-assembly strategies to understand photo-driven charge separation have had less emphasis.^3^,^13^–^16^

**Fig. 1:**
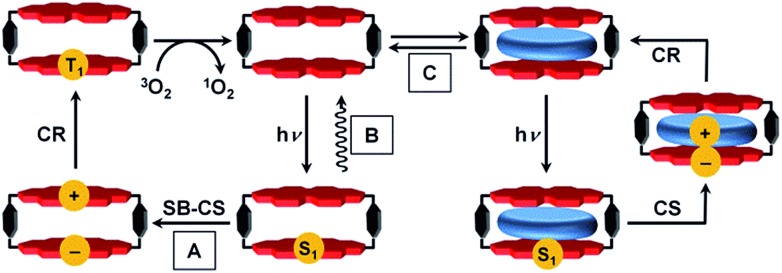
Schematic of the different excited state photophysics upon excitation; (A) symmetry-breaking charge separation and recombination to the PBI triplet within the PBI cyclophane **Cy-PBI**, in CH_2_Cl_2_ that can be used for singlet oxygen generation; (B) emission in toluene; (C) encapsulation of aromatic guests and photo-driven charge separation between guest and **Cy-PBI** and charge recombination to the ground state.

One factor thus far limiting studies of non-covalent supramolecular ensembles has been the lack of suitable dye-based hosts bearing sufficiently large cavities to complex redox-active guest molecules. In our recent work we approached this challenge with a cyclic perylene bisimide (PBI) trimer for which biomimetic intramolecular symmetry-breaking charge separation (SB-CS)^12^,^17^ could be observed in *τ*_CS_ = 12 ps, although the free energy of charge separation, Δ*G*_CS_, is barely negative.^17^ The charge recombination (CR) back to the PBI ground state (GS) is much slower (*τ*_CR_ = 1.12 ns), despite the larger Δ*G*_CR_ of this process. According to Marcus theory,^18^–^20^ ET can occur in the normal region (–Δ*G*_ET_ < *λ*) or in the inverted region (–Δ*G*_ET_ > *λ*) depending on the relative magnitudes of the Gibbs free energy (–Δ*G*_ET_) and the reorganization energy (*λ*).^6^,^21^,^22^ The PBI trimer data imply that its charge recombination reaction is in the Marcus inverted region.^18^–^20^,^23^,^24^ Unfortunately, no guest encapsulation could be achieved within the PBI trimer, in contrast to the PBI cyclophane, **Cy-PBI**, whose fluorescence is quenched by the encapsulation of electron rich aromatic guests.^25^ Accordingly, in the present study we elucidate the electronic interactions of **Cy-PBI** and its corresponding host–guest complexes by steady-state absorption, fluorescence and transient absorption (TA) spectroscopy to identify the ET processes and the individual lifetimes of the states formed. While **Cy-PBI** itself fluoresces strongly in low-polarity toluene, it undergoes intramolecular SB-CS followed by CR to the PBI triplet state upon excitation in polar solvents like CH_2_Cl_2_ (Fig. 1). Binding of electron-rich guests within **Cy-PBI** leads to intermolecular CS followed by CR back to the GS. Thus, the lowest excited singlet state of **Cy-PBI** decays in a complex sequence of events that can be modulated by solvent polarity or the presence of electron-rich guest molecules.

## Results and discussion

### Steady state spectroscopy and electrochemistry

**Fig. 2:**
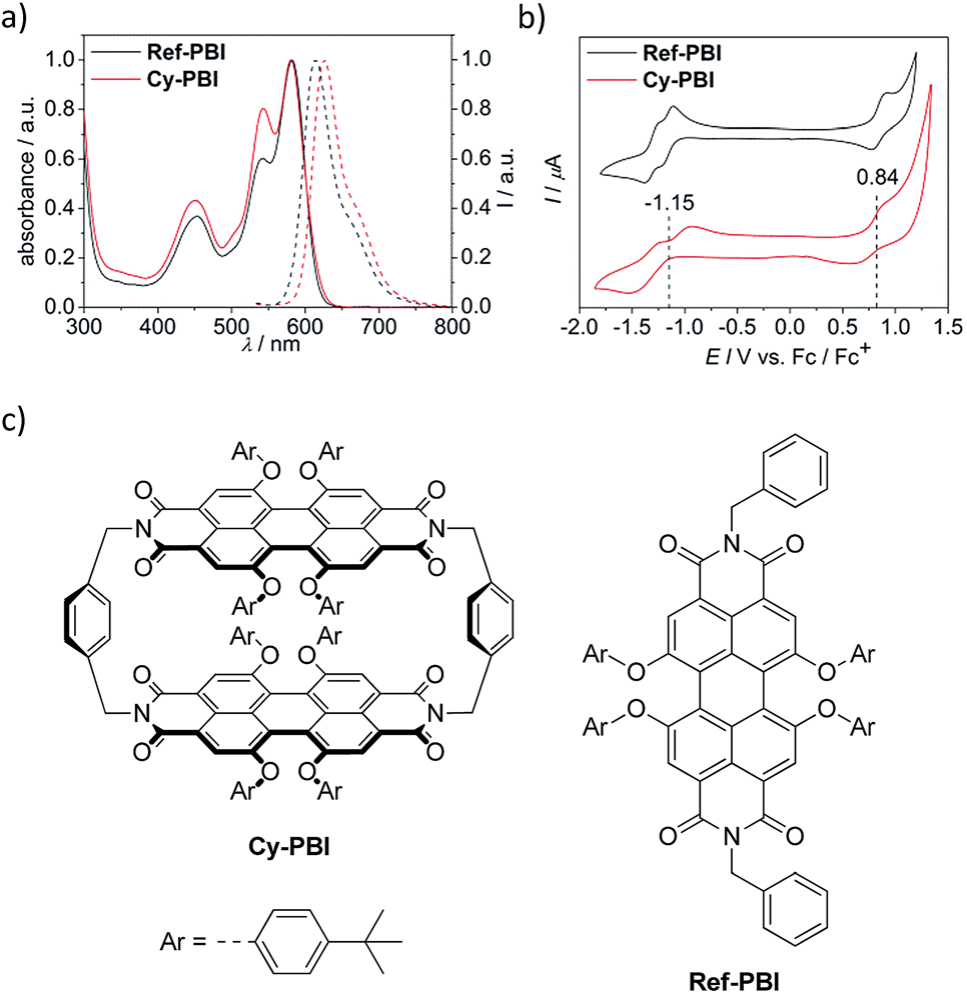
Normalized UV-vis absorption (solid line) and fluorescence (dashed line) spectra (a) of **Cy-PBI** and **Ref-PBI** (CH_2_Cl_2_, RT, *c* = 5 × 10^–5^ M); cyclic voltammograms (b) in CH_2_Cl_2_ (reference electrode: Ag/AgCl, working and auxiliary electrode: Pt; 0.1 M TBAHFP, Fc^+^/Fc, RT, *c* ∼ 2 × 10^–4^ M) and chemical structures (c) of **Cy-PBI** and **Ref-PBI**.

The steady-state UV-vis absorption spectrum of **Cy-PBI** has its maximum at 582 nm in CH_2_Cl_2_, which is comparable to the tetraphenoxy-substituted monomeric PBI (**Ref-PBI**). Similar to other multi-chromophoric PBI systems, the 0–1 vibronic band of **Cy-PBI** at 543 nm is significantly enhanced with respect to the 0–0 transition that can be related to excitonic coupling of the two cofacial PBI units (Fig. 2a).^17^,^26^,^27^ The corresponding fluorescence spectrum has its maximum at 627 nm, which is bathochromically shifted by 12 nm and has a significantly quenched 7% fluorescence quantum yield compared to **Ref-PBI** (*φ*_Fl_(**Ref-PBI**) = 75% in CH_2_Cl_2_), indicative of an efficient nonradiative decay process. No long-lived and red-shifted excimer-like emission^26^,^27^ was observed, which is attributed to the stiff linkage and the relatively long, 6.5 Å interplanar distance between the two PBI chromophores.

To calculate the free energy Δ*G*_CS_ we performed cyclic voltammetry on **Cy-PBI** and **Ref-PBI** in dry CH_2_Cl_2_ under argon (Fig. 2b). **Cy-PBI** shows similar behaviour to the monomer with two reversible reduction and one reversible oxidation waves, which are slightly cathodically shifted by 20 mV compared to **Ref-PBI**. The broadening of the **Cy-PBI** voltammogram can be related to multi-electron processes.^26^ To quantify Δ*G*_CS_ and Δ*G*_CR_ for the cyclophane we applied the Weller equation (eqn (1) and (2)):^28^1
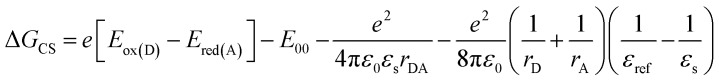

2 Δ*G*_CR_ = –(Δ*G*_CS_ + *E*_00_)

where *E*_ox(D)_ and *E*_red(A)_ are the first oxidation and reduction potentials of PBI, respectively, *E*_00_ is the excited state energy, *r*_DA_ is donor–acceptor center-to-center distance, and *r*_D_ and *r*_A_ are the effective ionic radii, respectively. The dielectric constants of the spectroscopic solvent and of the solvent used in electrochemistry are given with *ε*_s_ and *ε*_ref_. Since we used CH_2_Cl_2_ both for the spectroscopy and cyclic voltammetry studies, the Born ionic solvation energy (final) term in the Weller eqn (1) can be neglected. The oxidation and reduction potentials are 0.84 V and –1.15 V *vs.* Fc^+^/Fc, the excited state energy calculated from the average value of absorption and emission maxima of **Cy-PBI** is 2.06 eV, and the 0.65 nm distance between the two PBI units in **Cy-PBI** is obtained from the DFT calculated structure. Thus, the Gibbs free energy for intramolecular charge separation and recombination in **Cy-PBI** is calculated to Δ*G*_CS_ = –0.32 eV and Δ*G*_CR_ = –1.74 eV, respectively, confirming that both electron transfer processes are thermodynamically favorable.

### Transient absorption spectroscopy of the free host **Cy-PBI**

**Fig. 3:**
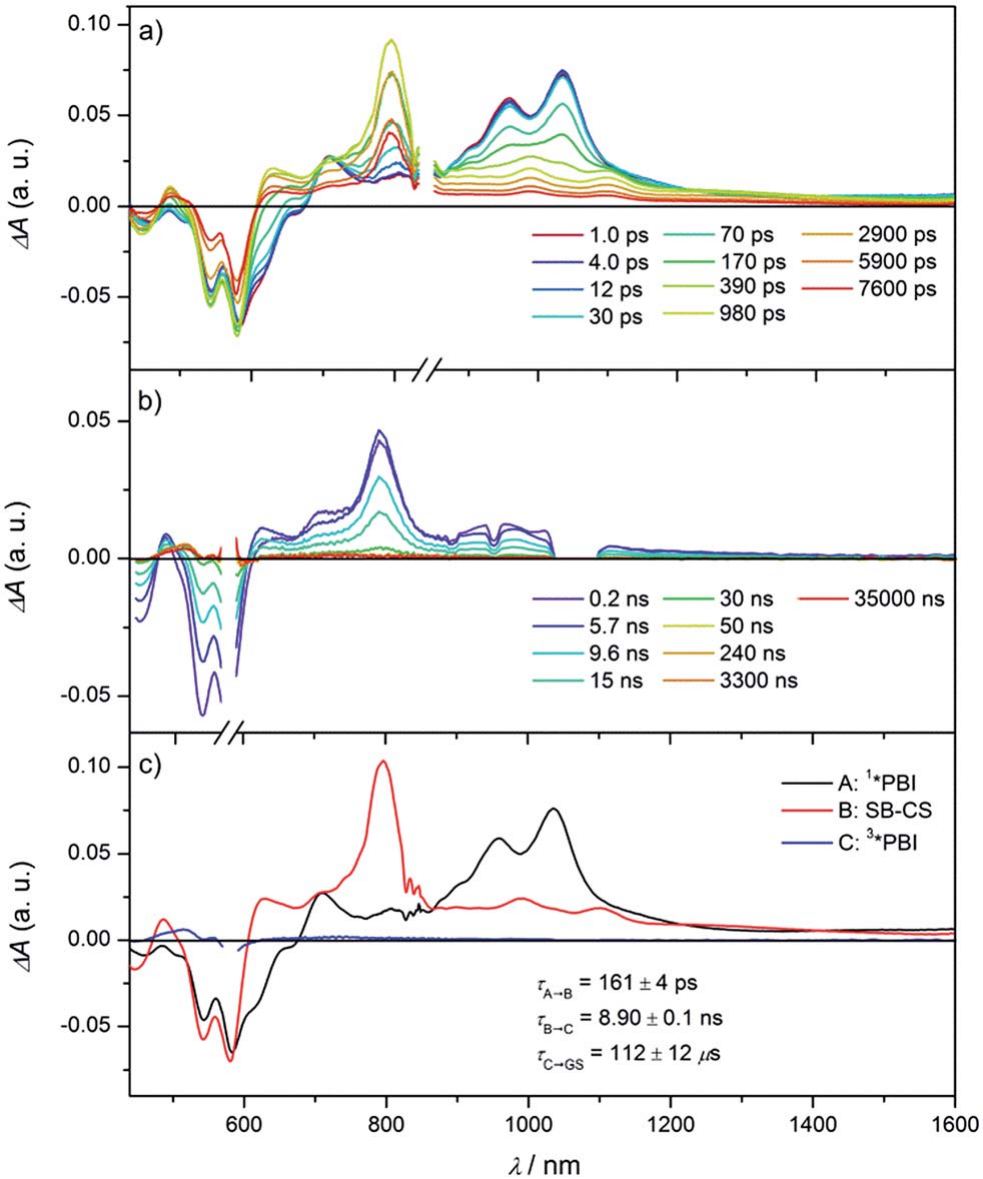
Femtosecond (a) and nanosecond (b) transient absorption spectra of **Cy-PBI** in CH_2_Cl_2_ showing the excited state dynamics after photoexcitation. Species-associated spectra (c) reconstructed from global fits to the sequential A → B → C → ground state model, where A is ^1^*PBI, B is SB-CS state and C is ^3^*PBI (*λ*_ex_ = 580 nm, 1.0 μJ per pulse, CH_2_Cl_2_, 298 K, degassed for ns TA). The C → ground state time was determined from ns TA.

To elucidate the excited state dynamics in **Cy-PBI**, we performed femtosecond (fs) and nanosecond (ns) TA studies (Fig. 3, S3 and S4† in the ESI†). The fs TA spectra (Fig. 3a) show the ground state bleach (GB) at 461, 543 and 583 nm and the stimulated emission (SE) at 611 and 664 nm as shoulders in the spectra. The excited singlet state ^1^*PBI absorption (ESA) has a positive signal at 710 nm and two characteristic strong maxima in the NIR region at 959 and 1035 nm. While the TA spectra and high fluorescence quantum yield for ^1^*Ref-PBI indicate that it decays back to the ground state primarily by emission (Fig. S2†), **Cy-PBI** shows very different excited state dynamics with a fast decay of the ^1^*PBI state in 161 ± 4 ps to a new transient species. Here the SE and ESA signals fully disappear, while new bands arise in the visible region at 486 and 628 nm, in the NIR region at 797, 993 and 1100 nm with a broad feature at ∼1220 nm.

By comparison to the PBI radical cation and anion absorption spectra these bands can clearly be attributed to PBI˙^+^ (486, 628, 1220 nm) and PBI˙^–^ (797, 993, 1100 nm).^17^,^29^ From these ultrafast transient dynamics data we conclude that photo-driven intramolecular SB-CS occurs in **Cy-PBI** with a high quantum yield.

Most interestingly and presumably caused by the long distance between the PBI moieties, CR between PBI˙^+^ and PBI˙^–^ in **Cy-PBI** occurs only slowly with 8.90 ± 0.06 ns to produce a significant yield of the PBI triplet state (^3^*PBI) characterized by positive absorptions at 515 and 556 nm, a bleach at 579 nm and a weak positive and broad absorption at 734 nm (Fig. 3b).^30^

### Triplet formation mechanism

Spin–orbit induced intersystem crossing (SO-ISC) is slow in PBIs, leading to very low triplet quantum yields for common PBIs (<1%).^31^–^33^ Since formation of PBI^+^˙–PBI^–^˙ is a prerequisite for populating ^3^*PBI, either spin–orbit charge transfer intersystem crossing (SOCT-ISC) or radical pair intersystem crossing (RP-ISC) are responsible for ^3^*PBI formation.^30^ The SOCT-ISC mechanism requires large changes in orbital angular momentum upon formation of PBI^+^˙–PBI^–^˙, which would require the π systems of the two PBI molecules to be nearly orthogonal,^34^–^36^ which is not the case in **Cy-PBI**. In contrast, the RP-ISC mechanism requires a relatively weak magnetic interaction between the two PBI radicals within PBI^+^˙–PBI^–^˙.^37^,^38^ Photoexcitation of **Cy-PBI** produces ^1^(PBI^+^˙–PBI^–^˙), whose spin dynamics depend strongly on the isotropic spin–spin exchange interaction, 2*J* = *E*_S_ – *E*_T_, where *E*_S_ and *E*_T_ are the energies of ^1^(PBI^+^˙–PBI^–^˙) and ^3^(PBI^+^˙–PBI^–^˙), respectively.^39^ Due to the long through-space and through-bond distances between the PBI subunits, 2*J* for PBI^+^˙–PBI^–^˙ should be small enough to enable RP-ISC of ^1^(PBI^+^˙–PBI^–^˙) to ^3^(PBI^+^˙–PBI^–^˙).^37^,^38^ Moreover, since 2*J* ∝ *V*^2^,^39^ and *k*_ET_ ∝ *V*^2^ (eqn (3)), the relatively long 8.90 ns CR time is also consistent with a small value of 2*J*. The subsequent CR process is spin selective in that ^1^(PBI^+^˙–PBI^–^˙) recombines back to the singlet ground state, whereas ^3^(PBI^+^˙–PBI^–^˙) recombines to ^3^*PBI within **Cy-PBI**.^40^ Our experimental findings, thus, indicate that RP-ISC is the most likely mechanism producing ^3^*PBI within **Cy-PBI**. Unfortunately, the 8.90 ns PBI^+^˙–PBI^–^˙ lifetime is too short to observe this RP directly by time-resolved EPR spectroscopy.

The lifetime of ^3^*PBI within **Cy-PBI** is very long (*τ* ≥ 112 μs) in a degassed solution at room temperature. Isolating **Cy-PBI** in a glassy solvent matrix is necessary to prohibit quenching by diffusion; however, this also prohibits the SB-CS process and thus, the intrinsic triplet lifetime could not be investigated. To estimate the quantum yield for the triplet formation, singlet oxygen emission was measured and compared to that of a methylene blue standard (MB).^41^ From this experiment, the singlet oxygen quantum yield, *φ*_Δ_ = 0.27, which also serves as the lower limit of the ^3^*PBI yield, and is consistent with the weak triplet signal in the TA spectra (Fig. 3c). This result and the low fluorescence quantum yield indicate that the main pathway back to the ground state is by singlet RP recombination. Furthermore, no photobleaching of **Cy-PBI** with singlet oxygen was observed, verifying the great photostability of PBIs against oxidation. The SB-CS process is disfavoured in non-polar solvents such as toluene as evidenced by the increase in **Cy-PBI** fluorescence quantum yield to 64%.^42^ Consistent with the increased emission, the transient absorption spectra of **Cy-PBI** in toluene show only singlet excited state decay directly back to the GS in *τ* = 4.5 ± 0.6 ns without the population of other transient species (Fig. S5†).

### Transient absorption spectroscopy of the host–guest complexes

By adding electron-rich guests, such as carbazole, pyrene, anthracene, and perylene to **Cy-PBI**, host–guest complexes are formed, leading to a slight bathochromic shift of the **Cy-PBI** absorption maximum and the appearance of a new band at longer wavelength that can be attributed to a charge transfer transition. Furthermore, the PBI fluorescence is almost fully quenched in the presence of these guests (Fig. S1†).^25^ The oxidation potentials of carbazole, pyrene, anthracene, and perylene are 0.64,^43^ 0.91,^44^ 0.88,^45^ and 0.59 V^46^*vs.* Fc^+^/Fc, respectively. Using these data, eqn (1), and the calculated 0.35 nm PBI–guest distance in the complex, we calculate Δ*G*_CS_ = –0.73, –0.46, –0.49 and –0.78 eV, respectively, and Δ*G*_CR_ = –1.33, –1.60, –1.57 and –1.28 eV for the host–guest complexes (guest@**Cy-PBI**), which clearly show that the electron transfer processes in the complexes are highly favored thermodynamically.

**Fig. 4:**
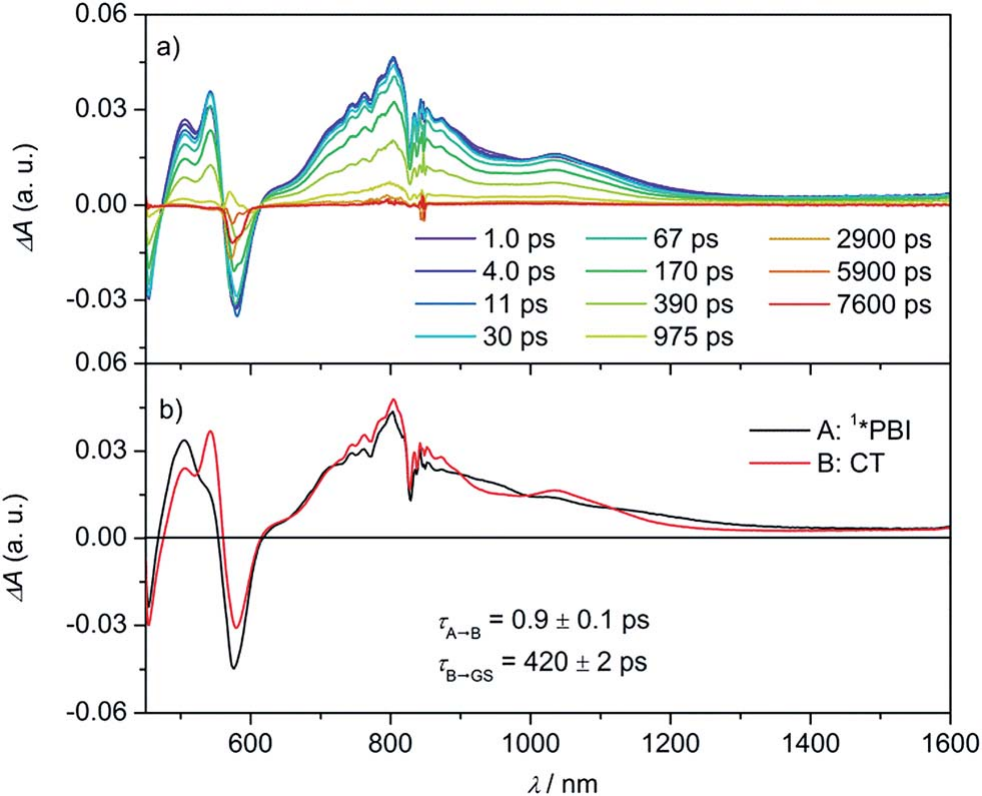
Femtosecond (a) transient absorption spectra of the perylene@**Cy-PBI** complex showing the excited state dynamics after photoexcitation. Species-associated spectrum (b) reconstructed from global fits to the sequential A → B → ground state (GS) model, where A is ^1^*PBI and B is CT state (*λ*_ex_ = 580 nm, 1.0 μJ per pulse, CH_2_Cl_2_, 298 K, air equilibrated).

In the fsTA spectra, an ultrafast CS components of *τ*_CS_ = 6.7 ± 0.2, 3.6 ± 0.3, 1.1 ± 0.2 and 0.9 ± 0.1 ps were observed for **Cy-PBI** complexed with carbazole, pyrene, anthracene and perylene, respectively (Fig. 4 and S6–S9†). The fsTA spectra of the perylene@**Cy-PBI** complex in Fig. 4 show the PBI^–^˙ absorptions along with a strong positive absorption at 542 nm that can be attributed to perylene^+^˙.^47^ The radical cation features of the other hydrocarbons are much weaker in the observed spectral window and strongly overlap with the GSB and PBI˙^–^ absorption changes, and were thus not observed. However, no PBI^+^˙ bands were detected, confirming that the CS now takes place between **Cy-PBI** and the guest molecule alone.

**Fig. 5:**
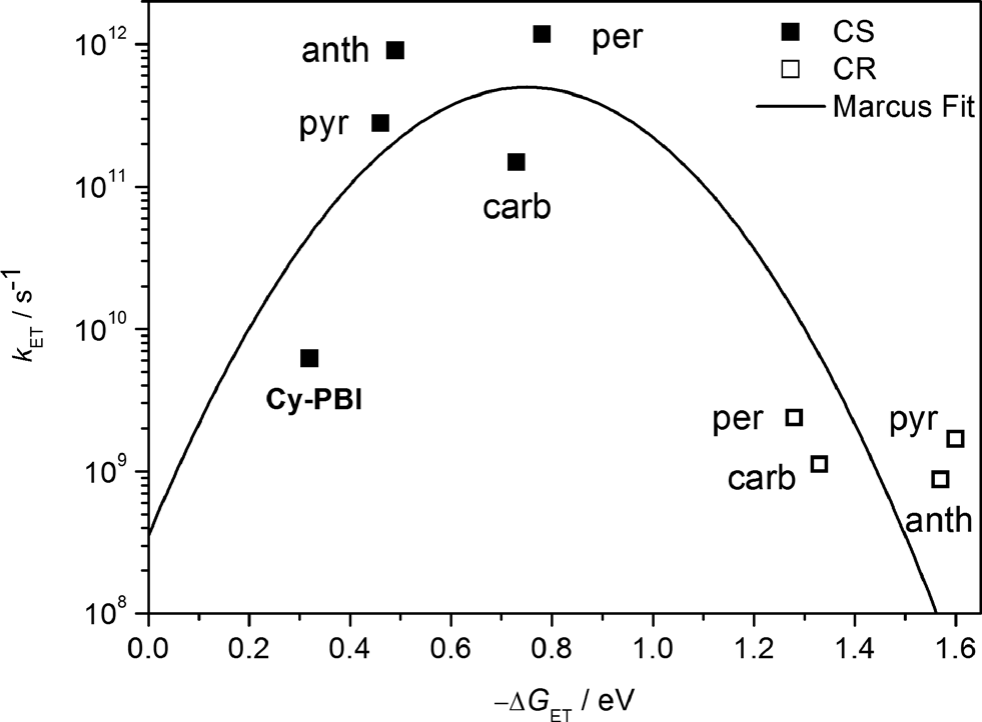
Plot of electron transfer rate constants in the PBI cyclophane and the corresponding host–guest complexes *vs.* the thermodynamic driving force (–Δ*G*_ET_) for charge separation (solid squares) and charge recombination (open squares); the line represents the fit according to eqn (3) with *λ* = 0.75 eV and *V* = 15 cm^–1^.

The ultrafast charge separation processes in the complexes are close to our detection limit (∼200 fs); thus the spectra of ^1^*PBI are difficult to observe for the perylene and anthracene complexes, but are clearer for pyrene and carbazole. The second time constant gives the CR lifetime directly back to the GS of PBI without any indication of triplet formation. This is consistent with a large 2*J* in the guest^+^˙–PBI˙^–^ RP, which precludes RP-ISC. The corresponding CR times using the carbazole, pyrene, anthracene and perylene guests are *τ*_CR_ = 892 ± 46, 593 ± 42, 1140 ± 120 to 420 ± 2 ps, respectively.

The data show that the CS rate increases with increasing Δ*G*_CS_ for ET from the HOMO of the respective electron-donating guest to the photo-excited electron-accepting PBI (Fig. 5). Using *τ*_CS_ and *τ*_CR_ obtained from the transient absorption kinetics and the corresponding values of Δ*G*_CS_ and Δ*G*_CR_, the total reorganization energy *λ* = *λ*_S_ + *λ*_I_, where *λ*_S_ and *λ*_I_ are the solvent and internal reorganization energies, respectively, and the electronic coupling matrix element *V* can be calculated according to Marcus theory by applying eqn (3):^19^3
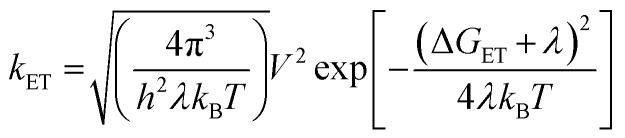
where *k*_ET_ is the electron transfer rate constant calculated from the transient absorption spectra, and Δ*G*_ET_ is the Gibbs free energy for charge separation or recombination. Given that the difference in *λ* between the GS and the excited singlet state for rigid aromatic molecules like PBI is very small, a single curve is drawn through both the CS and CR data, even though, strictly speaking, they represent two different reactions: (1) excited singlet state → RP and (2) RP → GS. The experimental data of *k*_ET_ were fitted by eqn (3), giving a reorganization energy *λ* = 0.75 eV and an electronic coupling matrix element *V* = 15 cm^–1^. The relatively high value of *λ* is consistent with a large *λ*_S_ resulting from reorientation of polar CH_2_Cl_2_ in response to the formation or decay of the RP charges.^7^ Furthermore, the data show that the CS lies in the Marcus normal region and reaches the peak of the Marcus parabola for the perylene@**Cy-PBI** complex, where –Δ*G*_ET_ ≅ *λ*. In contrast, the CR lies far in the Marcus inverted region, where the ET rates decrease with increasing free energy changes. The slow recombination observed in the Marcus inverted region is in general considered advantageous because long-lived charge separated states offer the possibility to utilize their stored energy for desired purposes such as artificial photosynthesis.^1^–^4^,^6^,^7^,^9^–^11^

## Conclusions

In summary, we have shown that the PBI cyclophane **Cy-PBI** undergoes intramolecular symmetry-breaking charge separation and slow charge recombination, which is accompanied by RP-ISC leading to ^3^*PBI that can be used to generate singlet oxygen with a 27% quantum yield.

Since ^3^*PBI is not accessible by conventional SO-ISC,^48^ the RP-ISC pathway to ^3^*PBI offers the possibility of developing an entirely new set of PBI applications, as demonstrated here with singlet oxygen generation. In contrast, the CS reaction within **Cy-PBI** is endergonic in a non-polar solvent like toluene, resulting in a high **Cy-PBI** fluorescence quantum yield. Binding electron-rich guest molecules within the **Cy-PBI** host affords a complete change of the photoexcited state relaxation pathway leading to intermolecular charge separation within a few picoseconds with formation of the radical cation of the guest and the PBI radical anion. Our findings show that the PBI cyclophane is indeed a special dye pair whose excited state properties are effectively modulated by its solvent environment as well as host–guest complex formation with electron donors.

## Experimental methods

### Synthesis

The tetraphenoxy-substituted perylene bisimide cyclophane (**Cy-PBI**) and the monomeric reference compound (**Ref-PBI**) were prepared according to literature.^25^

### Steady-state spectroscopy

UV-vis absorption spectroscopy was performed on a Perkin-Elmer Lambda 35 or Lambda 950 spectrometer. Solvents for spectroscopic studies were of spectroscopic grade and used without further purification. Fluorescence spectroscopy was performed on a PTI QM-4/2003 spectrofluorimeter. The fluorescence quantum yields were determined by optical dilution method^49^ (OD_max_ < 0.05) as the average value of four different excitation wavelengths using *N*,*N*′-(2,6-di-*iso*-propylphenyl)-1,6,7,12-tetraphenoxyperylen-3,4:9,10-tetracarboxylic acid bisimide (*φ*_fl_ = 0.96 in chloroform) as reference. Singlet oxygen emission was recorded on a PTI spectrofluorimeter. The quantum yield of singlet oxygen was determined in an air-equilibrated solution of **Cy-PBI** in dichloromethane (OD_max_ ∼ 0.5) as the average value of four different excitation wavelength using methylene blue as reference (*φ*_Δ_ = 0.57 in dichloromethane).^41^

### Electrochemistry

Cyclic voltammetry was carried out with a standard commercial electrochemical analyzer (EC epsilon; BAS Instruments, UK) in a three electrode single-compartment cell. The supporting electrolyte tetrabutylammonium hexafluorophosphate (TBAHFP) was recrystallized from ethanol/water. As an internal standard for the calibration of the potential ferrocenium/ferrocene (Fc^+^/Fc) was used. As reference electrode Ag/AgCl and as working and auxiliary electrodes a Pt disc and a Pt wire were used.

### Transient absorption spectroscopy

Femtosecond and nanosecond transient absorption experiments were performed using an instrument as previously described^50^ with an approximately 120 fs output of a commercial Ti:sapphire oscillator/amplifier (Tsunami/Spitfire, Spectra-Physics) that was split to seed and pump a laboratory-constructed optical parametric amplifier used to generate the 569 nm excitation (“pump”) beam and a femtosecond continuum probe, by using a 3 mm sapphire plate for the visible range or a proprietary crystal for the near-infrared (NIR) spectral region (Ultrafast Systems, LLC). Transient spectra were collected by using customized commercial detectors (Helios, Ultrafast Systems, LLC). Experiments were performed with a depolarized pump to eliminate contributions from orientational dynamics. The kinetic analysis is based on a global fit to selected single-wavelength kinetics. Several kinetic traces at different wavelengths were chosen and fitted globally to a kinetic model. The differential equations were solved and then convoluted with the instrument response function, before employing a least-square fitting to find the parameters which result in matches to the same functions for all selected wavelengths (MATLAB). These parameters are then fed directly into the differential equations, which were solved for the populations of the states in model. Finally, the raw data matrix (with all the raw data) is deconvoluted with the populations as functions of time to produce the species-associated spectra.

### Molecular modelling

DFT calculations were performed by using the Gaussian 09 program package^51^ with B3-LYP^52^–^54^ as functional and 6-31+G* as basis set. The structures were geometry optimized, followed by frequency calculations on the optimized structures which confirmed the existence of a minimum (one very small imaginary frequency of 4i cm^–1^ was obtained for **Cy-PBI**. Small imaginary frequencies (<100i cm^–1^) are considered most likely to be an artefact of the calculation instead of an indication of a transition state^55^).

## Supplementary Material

Supplementary information
