# Supplementary material for: Guest and solvent modulated photo-driven charge separation and triplet generation in a perylene bisimide cyclophane
Source: Chem Sci. 2016 May 18;7(8):5428–34. doi: 10.1039/c6sc01574c (PMC6021749; doi:10.1039/c6sc01574c)
Supplement: Supplementary file 1 [file SC-007-C6SC01574C-s001.pdf]

Electronic Supplementary Material (ESI) for Chemical Science.
This journal is © The Royal Society of Chemistry 2016

Electronic Supplementary Information for

Guest and solvent modulated photo-driven charge separation and triplet generation in a perylene bisimide cyclophane

Peter Spenst,[a] Ryan M. Young,[b] Michael R. Wasielewski*[b] and Frank Würthner*[a]

[a] Institut für Organische Chemie and Center for Nanosystems Chemistry

Universität Würzburg, Am Hubland, 97074 Würzburg, Germany

E-mail: wuerthner@chemie.uni-wuerzburg.de

[b] Department of Chemistry and Argonne-Northwestern Solar Energy Research (ANSER) Center

Northwestern University, 2145 Sheridan Road, Evanston, IL 60208-3113 (USA)

E-mail: m-wasielewski@northwestern.edu

Table of Contents

1. Optical Spectroscopy 2

2. Transient Absorption Spectroscopy 3

1. Optical spectroscopy

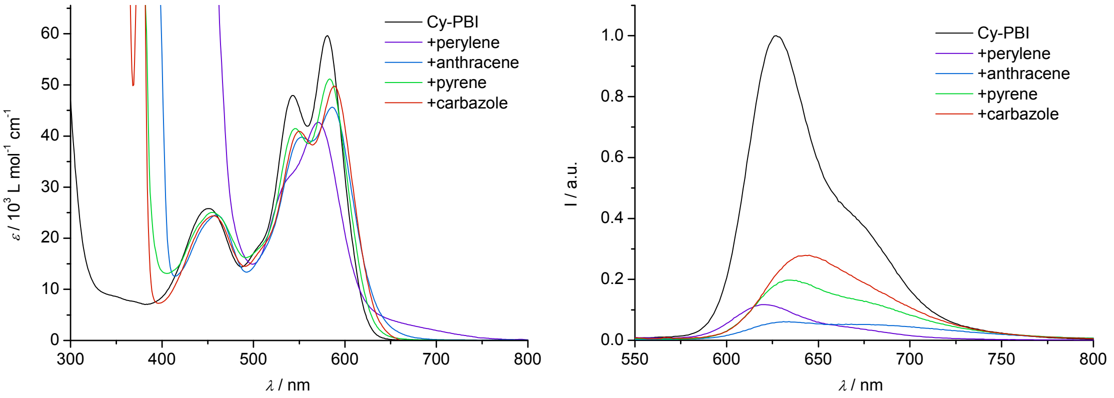

Fig. S1 UV-vis absorption (left) and fluorescence (right) spectra of **Cy-PBI** and guest@**Cy-PBI** in dichloromethane; *c*(**Cy-PBI**) = 5 x 10^{-6} M, RT.

2. Transient absorption spectroscopy

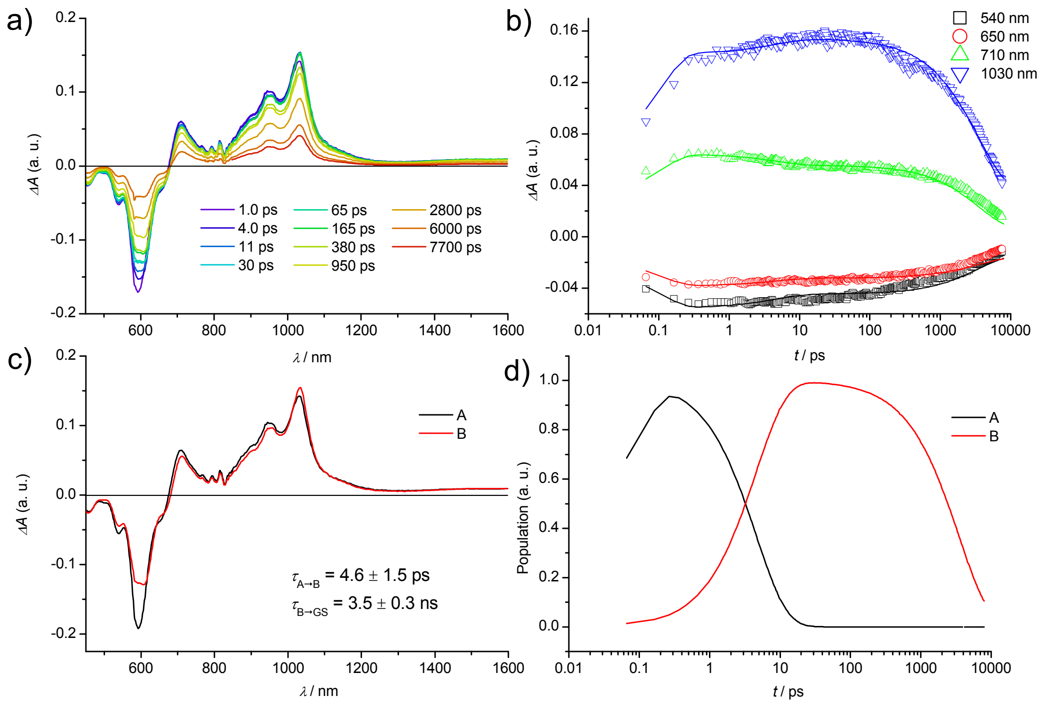

Fig. S2 a) Femtosecond transient absorption of **Ref-PBI** in dichloromethane showing excited state dynamics after photoexcitation; b) plots of selected kinetic traces superimposed with matching curves at different wavelengths; c) species-associated spectra plots; d) kinetic model plots (λ_{ex} = 580 nm, 1.0 μJ/pulse, 298 K). A and B: singlet excited state S_1 (A→B likely presents relaxation on the singlet state based on the timescale and the similarity of the spectra).

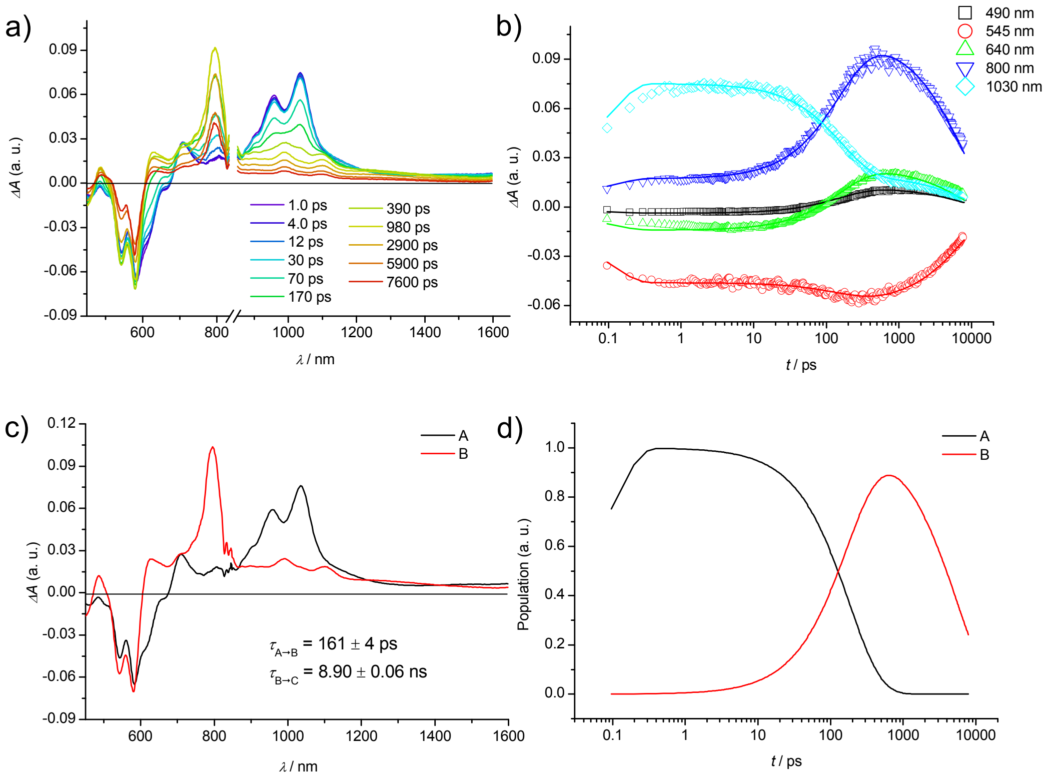

Fig. S3 a) Femtosecond transient absorption of the **Cy-PBI** in dichloromethane showing excited state dynamics after photoexcitation; b) plots of selected kinetic traces superimposed with matching curves at different wavelengths; c) species-associated spectra plots; d) kinetic model plots (λ_{ex} = 580 nm, 1.0 μJ/pulse, 298 K). A: singlet excited state S_1; B: charge separated state SB-CS.

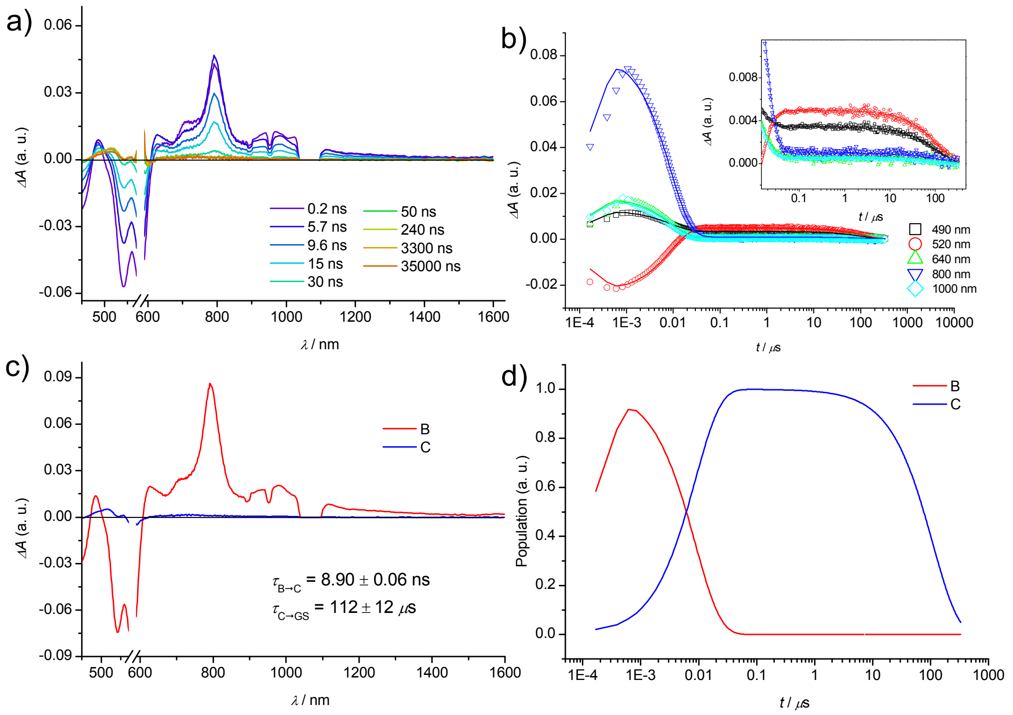

Fig. S4 a) Nanosecond transient absorption of **Cy-PBI** in dichloromethane showing excited state dynamics after photoexcitation; b) plots of selected kinetic traces superimposed with matching curves at different wavelength; c) species-associated spectra plots; d) kinetic model plots (λ_{ex} = 580 nm, 1.0 μJ/pulse, 298 K). B: charge separated state SB-CS; C: PBI triplet state.

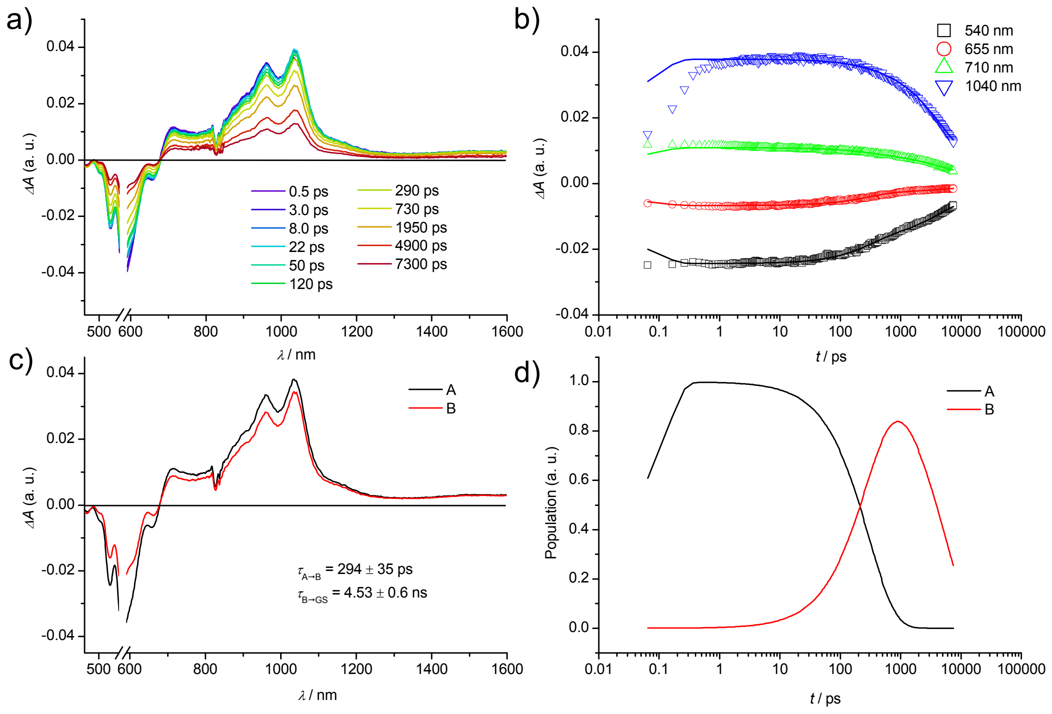

Fig. S5 a) Femtosecond transient absorption of **Cy-PBI** in toluene showing excited state dynamics after photoexcitation; b) plots of selected kinetic traces superimposed with matching curves at different wavelengths; c) species-associated spectra plots; d) kinetic model plots (λ_{ex} = 580 nm, 1.0 μJ/pulse, 298 K). A and B: singlet excited state S_1 (A→B likely presents relaxation on the singlet state based on the timescale and the similarity of the spectra).

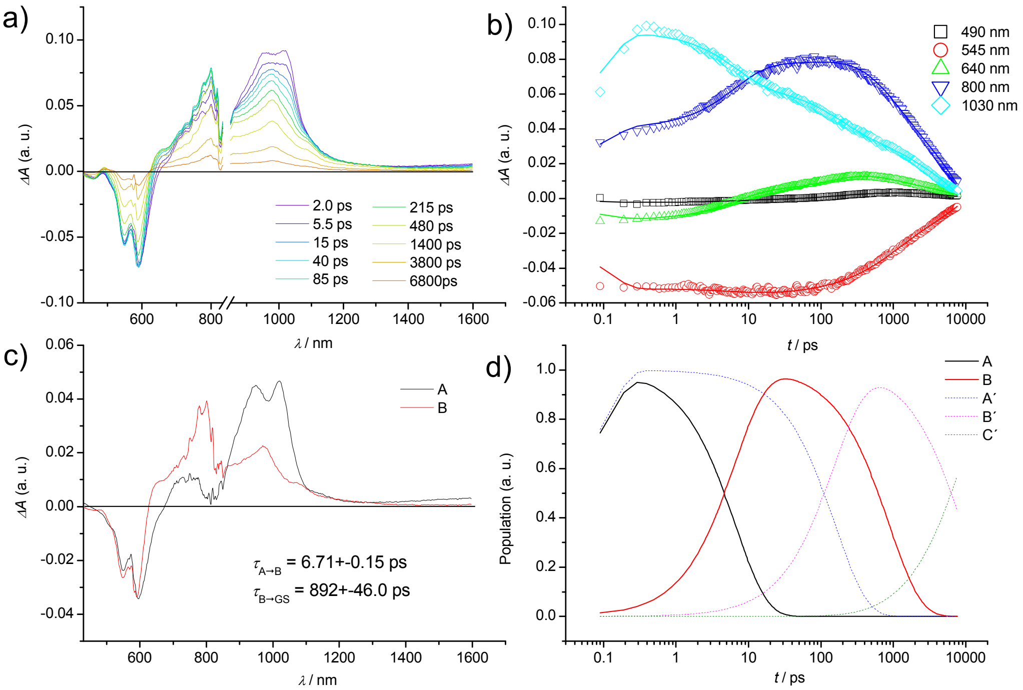

Fig. S6 a) Femtosecond transient absorption of the carbazole@**Cy-PBI** complex in dichloromethane showing excited state dynamics after photoexcitation; b) plots of selected kinetic traces superimposed with matching curves at different wavelength; c) species-associated spectra plots; d) kinetic model plots (λ_{ex} = 580 nm, 1.0 μJ/pulse, 298 K). A: singlet excited state S_1; B: charge transfer state CT; A´, B´ and C´ correspond to the free **Cy-PBI** and are not shown in c) for clarity reasons.

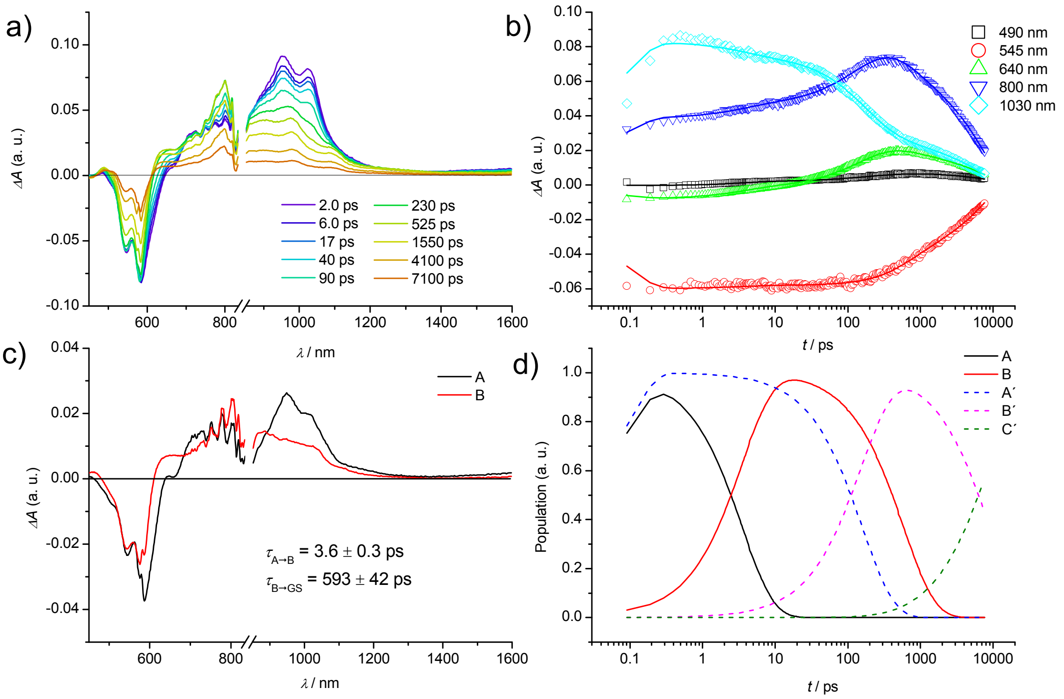

Fig. S7 a) Femtosecond transient absorption of the pyrene@ **Cy-PBI** complex in dichloromethane showing excited state dynamics after photoexcitation; b) plots of selected kinetic traces superimposed with matching curves at different wavelength; c) species-associated spectra plots; d) kinetic model plots (λ_{ex} = 580 nm, 1.0 μJ/pulse, 298 K). A: singlet excited state S_1; B: charge transfer state CT; A´, B´ and C´ correspond to the free **Cy-PBI** and are not shown in c) for clarity reasons.

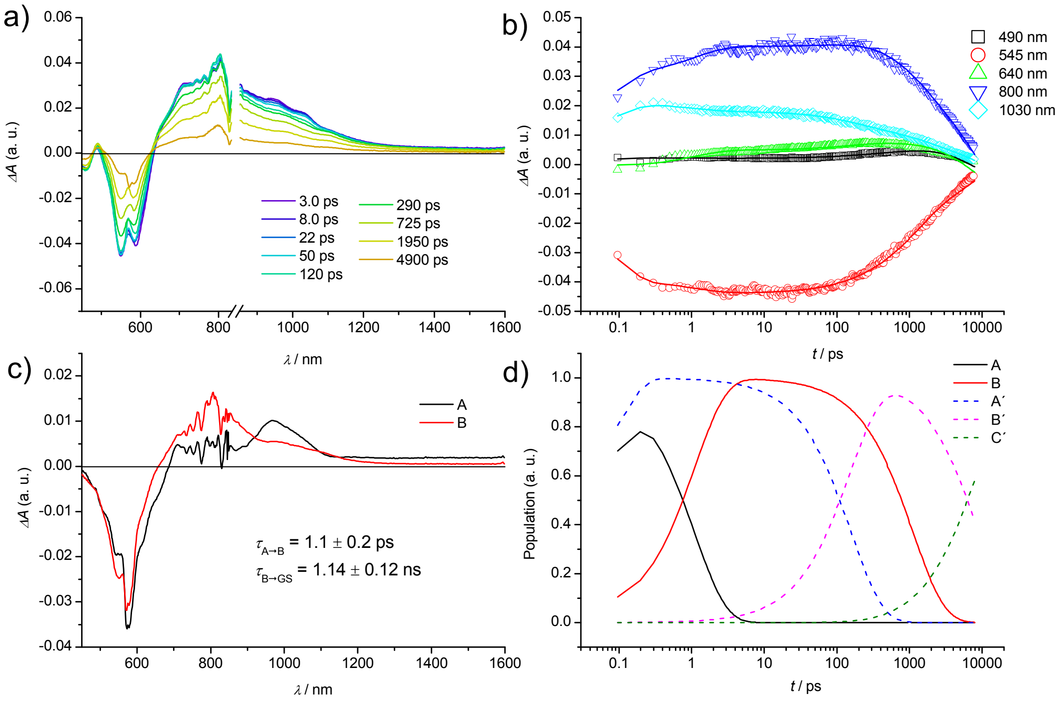

Fig. S8 a) Femtosecond transient absorption of the anthracene@**Cy-PBI** complex in dichloromethane showing excited state dynamics after photoexcitation; b) plots of selected kinetic traces superimposed with matching curves at different wavelength; c) species-associated spectra plots; d) kinetic model plots (λ_{ex} = 580 nm, 1.0 μJ/pulse, 298 K). A: singlet excited state S_1; B: charge transfer state CT; A´, B´ and C´ correspond to the free **Cy-PBI** and are not shown in c) for clarity reasons.

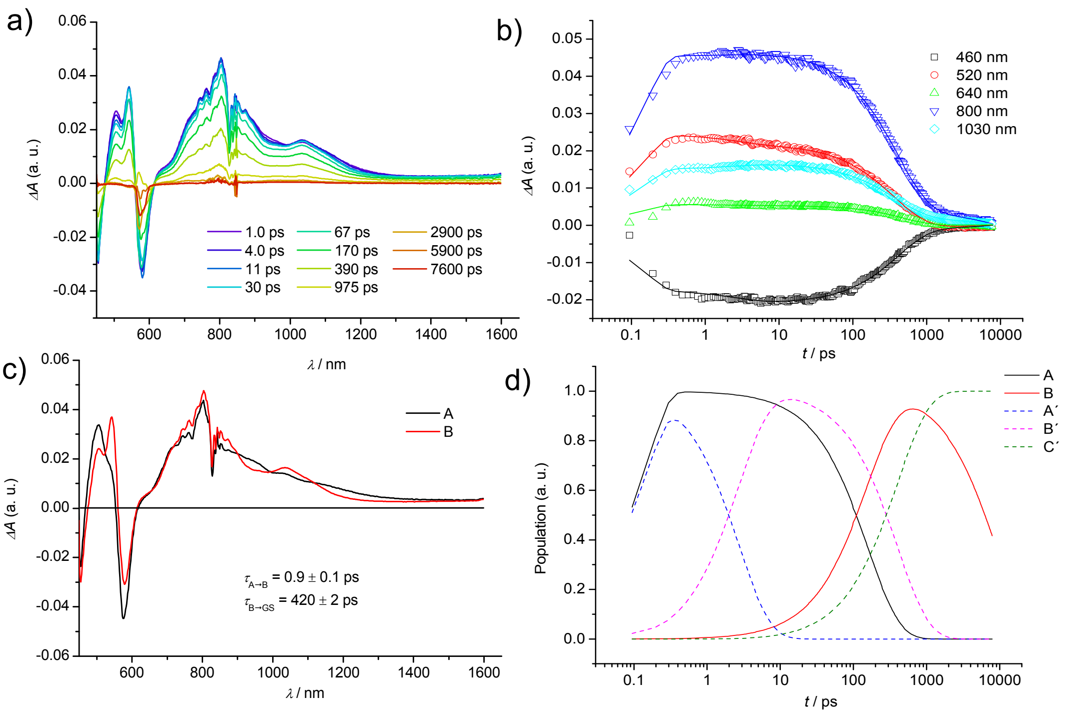

Fig. S9 a) Femtosecond transient absorption of the perylene@**Cy-PBI** complex in dichloromethane showing excited state dynamics after photoexcitation; b) plots of selected kinetic traces superimposed with matching curves at different wavelength; c) species-associated spectra plots; d) kinetic model plots (λ_{ex} = 580 nm, 1.0 μJ/pulse, 298 K). A: singlet excited state S_1; B: charge transfer state CT; A´, B´ and C´ correspond to the free **Cy-PBI** and are not shown in c) for clarity reasons.
